# Acute coronary syndrome risk prediction based on gradient boosted tree feature selection and recursive feature elimination: A dataset-specific modeling study

**DOI:** 10.1371/journal.pone.0278217

**Published:** 2022-11-29

**Authors:** Huizhong Lin, Yutao Xue, Kaizhi Chen, Shangping Zhong, Lianglong Chen

**Affiliations:** 1 Department of Cardiology, Fujian Heart Medical Center, Fujian Institute of Coronary Heart Disease, Fujian Medical University Union Hospital, Fuzhou, PR China; 2 College of Computer and Data Science, Fuzhou University, Fujian, China; Victoria University, AUSTRALIA

## Abstract

Acute coronary syndrome (ACS) is a serious cardiovascular disease that can lead to cardiac arrest if not diagnosed promptly. However, in the actual diagnosis and treatment of ACS, there will be a large number of redundant related features that interfere with the judgment of professionals. Further, existing methods have difficulty identifying high-quality ACS features from these data, and the interpretability work is insufficient. In response to this problem, this paper uses a hybrid feature selection method based on gradient boosting trees and recursive feature elimination with cross-validation (RFECV) to reduce ACS feature redundancy and uses interpretable feature learning for feature selection to retain the most discriminative features. While reducing the feature set search space, this method can balance model simplicity and learning performance to select the best feature subset. We leverage the interpretability of gradient boosting trees to aid in understanding key features of ACS, linking the eigenvalue meaning of instances to model risk predictions to provide interpretability for the classifier. The data set used in this paper is patient records after percutaneous coronary intervention (PCI) in a tertiary hospital in Fujian Province, China from 2016 to 2021. In this paper, we experimentally explored the impact of our method on ACS risk prediction. We extracted 25 key variables from 430 complex ACS medical features, with a feature reduction rate of 94.19%, and identified 5 key ACS factors. Compared with different baseline methods (Logistic Regression, Random Forest, Gradient Boosting, Extreme Gradient Boosting, Multilayer Perceptron, and 1D Convolutional Networks), the results show that our method achieves the highest Accuracy of 98.8%.

## 1. Introduction

The World Health Organization reports that more than 12 million people worldwide die from cardiovascular problems. Cardiovascular disease (CVD) is very common in China. According to relevant data, the number of people suffering from cardiovascular disease in China is approximately 290 million [1], with more than 2 million new patients every year and a high mortality rate. It ranks first in cancer and other diseases. According to incomplete statistics, the medical cost of cardiovascular diseases in China has reached $2.86 billion, and the high treatment cost has put a serious medical burden on patients. Acute coronary syndrome (ACS), a frequent but serious form of coronary artery disease [2, 3], is distinguished by primary atherosclerotic plaque rupture and secondary completely or partially occlusive thrombus that cause ST-segment elevation myocardial infarction (STEMI), non-STEMI, and unstable angina (UA) [4]. Not only is there a high mortality rate, but patients are also at an equally high risk of recurrence after discharge from the first treatment, both of which are drivers of poor prognosis.

Relevant studies have shown [5] that when fat or harmful cholesterol accumulates in the arterial wall, the arterial wall is narrowed and eventually blocked. ACS depends on the acuteness and severity of coronary occlusion. Risk factors associated with ACS can be a combination of controllable factors (e.g., lifestyle) and uncontrollable factors (e.g., age, gender, family history, etc.). At present, the clinical methods used for ACS detection mainly include [6]: electrocardiogram, dynamic electrocardiogram, hematology examination, CT angiography, etc. These inspection methods are limited to a certain extent by the doctor’s personal subjective judgment and long-term experience. A thorough evaluation of these risks is crucial to the clinical management of each patient’s health and offers alternatives to the best secondary preventive medications.

Establishing an appropriate disease risk assessment model is a critical step in ACS risk assessment and subsequent management decisions. Major adverse cardiovascular events (MACE) prediction has been widely used in the early prevention and intervention of ACS patients [7, 8], and it is an important tool to assess the likelihood of ACS risk. Similar to the MACE practice, the current risk scoring tools with high awareness in clinical medicine also include the Global Registry of Acute Coronary Events (GRACE) [9], Framingham Risk Score [10], etc., which are also often used to evaluate ACS patients’ severity. However, these risk scores have some limitations, such as only considering prognostic factors in a subset of patients, and none of the patients received the current standard of care. In addition, prior standard risk score methods were unable to accurately predict outcomes for ACS patients [9, 11]. Therefore, it is necessary to reconsider new methods to effectively predict the risk of ACS patients.

In recent years, machine learning has performed well for ACS prediction [11], and can significantly improve the performance. Various machine learning modeling methods, including Naive Bayes, Random Forest, Gradient Boosting, Logistic Regression, and Support Vector Machines, have been employed in numerous studies for the diagnosis and risk assessment of ACS [12–14]. Machine learning can also mine potential risk factors for disease. In the study of [15], by building a coronary heart disease prediction model based on a decision tree algorithm, it was found that an important variable for coronary heart disease is the serum hs-CRP level. PRAISE [16] is a machine learning-based risk stratification model that combines clinical, anatomical, and procedural features to predict all-cause mortality, recurrent acute myocardial infarction, and major bleeding in patients with ACS. But they almost all ignore the use of effective feature selection methods to filter the high-dimensional features of the original data.

Currently, there are still the following challenges in actual ACS risk prediction: (1) there are a large number of missing values, irrelevant and redundant features in the original ACS dataset, which may negatively affect the model training process [17]; (2) there are many risk factors that lead to ACS, but there is no effective feature selection method to identify these factors; (3) poor interpretability is also one of the challenges [18], especially for deep learning models; and (4) the experimental data faces the problem of imbalance, which affects the research.

To address the above challenges, the main contributions of this paper are summarized as follows:

1. We use a new ACS dataset from a hospital in Fujian Province, China, and perform a lot of complex data cleaning, data extraction, missing values, and other preprocessing work.
2. We used a hybrid (interpretable gradient boosting tree model + cross-validated RFE) feature selection method to screen out key features of ACS, which helped us discover important prognostic factors. This fusion method a) overcomes the problems of unstable feature selection and random division of nodes in gradient boosting trees; b). Compared with the direct use of the RFECV method, it improves the execution efficiency.
3. We statistically analyzed the high correlations between variables in the experimental data and used XGBoost, GB, and RF model feature importance to visualize the contribution of key features of ACS to interpretability.
4. We use a hybrid sampling SMOTETomek approach to improve model performance for predicting ACS risk in imbalanced datasets and compare it to other sampling techniques in experiments.

The structure of the paper is as follows: Section 2 introduces the related work of applying machine learning to predict ACS and feature selection in the medical field. Section 3 introduces our method. Section 4 presents the experimental data and results. Section 5 discusses the results. Finally, Section 6 presents the conclusion.

## 2. Related work

### 2.1 Disease prediction using machine learning

In the past decade, some medical organizations and institutions have studied disease prediction models based on machine learning (ML) methods. Machine learning is a discipline that uses intelligent techniques to learn predictive and descriptive models from data [19]. ML methods can perform risk assessment and prediction of diseases based on clinically abnormal data, and they can accurately find hidden effects in data instead of manual work. For ML methods, the conventional approach is to extract features and train a predictive model on them to automatically classify patients. Giri et al. [20] used the discrete wavelet transform to decompose the heart rate signal and applied principal component analysis, linear discriminant analysis, and independent component analysis to the wavelet coefficient set to reduce the data dimension. Then they use the support vector machine, Gaussian mixture model, probabilistic neural network, and K-nearest neighbor four classifiers to identify patients with coronary heart disease and normal people; Alickovic et al. [21] used an autoregressive model to extract features from ECG data, using K-nearest neighbors, support vector machines, multilayer perceptrons, and the radial basis function network to distinguish arrhythmia patients from normal people; For an automatic diagnosis system for Parkinson’s disease, Lamba et al. [22] used four transfer learning architectures: ResNet, DensNet, VGG, and AlexNet to classify spiral images of trainee populations; Kumar et al. [23] systematically introduced a decision support system (DSS) for diagnosing cardiac disease, analyzing various current problems and challenges in predicting cardiac disease.

### 2.2 Strategy for feature selection

Many studies have focused on providing new mechanisms to improve the performance of ACS modeling. Among them, feature selection (FS) is one of the most effective ways to improve model performance because it can mitigate the effects of noise and redundant variables. Feature selection focuses on selecting a subset of variables from the input multidimensional features that can effectively describe the input data while reducing the influence of noise or irrelevant variables. Therefore, it can improve model performance, reduce computational requirements, and better understand the goals of the data [24]. Among them, Rani et al. [25] used clinical data to diagnose coronary artery disease (CAD) and used the Extra Tree feature selection method to select relevant features. Zhang et al. [26] proposed a feature selection method for Holter by introducing the ovo combination method and using a support vector machine classifier to select an effective feature subset. This method has been used for heartbeat classification of ECG data.

In recent years, relevant scholars have also studied the hybrid feature selection method, which can usually combine the advantages of the two feature selection methods. Rani et al. [27] proposed a novel Hybrid Pearson Correlation and Backward Elimination (HPCBE) feature selection method, which achieved a feature reduction rate of over 50% in heart disease diagnosis. In [28], Rani et al. then proposed a hybrid feature selection method called CFGA that fuses CFS (Correlated Feature Selection) and GA (Genetic Algorithm), which has the advantage that it can be coupled with any classifier. Lamba et al. [29] proposed a hybrid MIRFE feature selection method for Parkinson’s disease patient classification.

## 3. Materials and methods

### 3.1 Data source

The dataset used in this study was real hospital patient data, and the data was partly provided by the Department of Cardiovascular Medicine of a tertiary hospital in Fujian Province, China. The dataset includes data on ACS patients collected through follow-up visits during the five-year period from 2016 to 2021. This included 5,850 patients who were discharged from the hospital after undergoing surgery (coronary angiography and revascularization PCI). Each patient had 430 records of various indicators, for a total of approximately 2,515,500 records. But the actual dataset contains a large number of missing patient records and the data is noisy and irregular, so the actual valid records are much lower than this number. We conducted this study in August 2021. The dataset consists mainly of structured and unstructured text data. Structured data includes basic information such as the patient’s age, gender, and living habits. Unstructured text data includes patients’ ECG examinations, doctors’ diagnostic records, and surgical operation records. In general, the content of the data set can be divided into seven categories: basic patient information; past medical history; electrocardiogram indicators; cardiac color Doppler ultrasound indicators; blood test indicators; medication status; and coronary vascular lesions. The clinical and therapeutic characteristics of the study population are presented in Table 1.

**Table 1:** Clinical characteristics of the experimental dataset.

Characteristics		Descriptive statistics			
		All(N = 2702)	Alive (N = 2582)	Death(N = 120)	p-value
**Basic clinical variables**					
Age (years)		65(57–72)	65(57–71)	72(64–78)	<0.001*
Gender	Female	495(18.3%)	455(17.6%)	40(33.3%)	<0.001*
	Male	2207(81.7%)	2127(82.4%)	80(66.7%)	<0.001*
BMI		24.0(22.7–26.0)	24.0(22.7–26.1)	24.0(22.8–24.6)	0.026
Smoking		1171(43.3%)	1129 (43.9%)	42(38.5%)	0.343
**Past medical history**					
Diabetes		794 (30.0%)	748 (29.4%)	46(42.6%)	0.001
Hypertension		1542 (57.5%)	1473 (57.2%)	69(65.1%)	0.179
History of renal insufficiency		26 (1.0%)	20 (0.8%)	6(5.7%)	<0.001*
**ECG**					
Heart rate (bpm)		68(61–76)	68(61–76)	75(60–86)	0.042
**Heart**					
E ’wave rate		0.06(0.04–0.07)	0.06(0.04–0.07)	0.03(0–0.05)	0.610
LV ejection fraction		63.3(55.1–68.6)	63.5(55.7–68.8)	38.1(0–61.0)	<0.001*
LV mass index		103.4(86.5–121.0)	113.8(87.4–121.0)	32(0–117.2)	0.005
**Blood test index**					
Total cholesterol		3.95(3.24–4.85)	3.97(3.25–4.88)	3.46(2.07–4.32)	0.674
LDL cholesterol		2.48(1.85–3.28)	2.50(1.88–3.29)	2.10(1.02–2.85)	0.226
Triglycerides		1.43(1.03–2.00)	1.44(1.04–2.01)	1.09(0.72–1.77)	0.794
NT-proBNP		129(37–538)	125(37–486)	641(9–3531)	0.988
Apolipoprotein A		1.19(1.04–1.33)	1.20(1.05–1.34)	0.94(0.70–1.19)	0.692
**Medical therapy**					
Statins		2099 (78.6%)	2046 (79.5%)	53(55.8%)	<0.001*
Spironolactone		252 (9.6%)	234 (9.2%)	18(19.8%)	<0.001*
Aspirin		2617 (98.2%)	2528 (98.3%)	89(95.7%)	<0.001*
**Coronary angiopathy variables**					
Bifurcation position		100(4.1%)	96(4.1%)	4(4.0%)	0.391
CTO		128(4.9%)	119(4.8%)	9(8.1%)	0.015
Angulation		3(0.1%)	3(0.1%)	0(0%)	<0.001*
Calcification		20(0.8%)	20(0.8%)	0(0%)	0.011
Lesion type		2610(96.6%)	2501(96.9%)	109(90.8%)	0.272
Target vessel		2640(97.7%)	2528(97.9%)	112(93.3%)	0.001

**Note:** The above structured data are all cases of ’yes’. BMI: body mass index; LV: left ventricle; NT-proBNP: N-terminal of the prohormone brain natriuretic peptide; ECG: electrocardiogram; LDL: low density lipoprotein. An asterisk (*) with a p-value less than 0.001 indicates a statistically significant difference in the variable between the survival and death groups. The information in the table is presented as n (%) and median value (IQR). The qualitative indicators of the data are expressed as the proportion of the population (missing values are not counted), and the quantitative indicators are expressed as the median and the first quartile (Q1) and third quartile (Q3) of the data (25–75%).

### 3.2 Outcomes

The results of this paper are intended to predict the likely outcome (death from all causes) in patients with ACS. The records of all-cause death are the ACS patients in our hospital who were followed up within 1 year after surgery (Table 2). These include cardiac and non-cardiac deaths. Before data preprocessing, 139 (2.4%) of 5764 patients died and 5625 (97.6%) patients were healthy within 1 year of follow-up; after data preprocessing, 120 (4.4%) of 2702 patients died and 2582 (95.6%) patients were healthy. The dataset is divided into two parts, and Table 3 shows the division of different cohorts: The training (60%) cohort, which is used to train six machine learning models and adjust their parameters; The test (40%) cohort, which is used to test unknown data generalization ability and evaluation performance.

**Table 2:** Patient outcomes.

Outcome	Variable	Before preprocessing (N = 5764)	After preprocessing (N = 2702)
All-cause mortality	alive	5625(97.6%)	2582(95.6%)
	death	139(2.4%)	120(4.4)
	feature dimension	87(20%)	25(5.8%)

**Table 3:** Division of training queue and test queue.

Queue	Variable	Number	Proportion
Training set (60%)	alive	1552	95.7%
	death	69	4.3%
Test set (40%)	alive	1030	95.3%
	death	51	4.7%

### 3.3 Risk factors

Ultimately, clinical features with high risk factors for ACS included 25 variables: among them clinical variables (sex, age, BMI, smoking); medical history variables (diabetes, hypertension, history of renal insufficiency); electrocardiographic variables (heart rate); cardiac ultrasound variables (e’ wave velocity, left ventricular ejection fraction, left ventricular weight index); blood test index variables (low density lipoprotein, total cholesterol, triglyceride, NT-proBNP, apolipoprotein A); medication variables (statin, spironolactone, aspirin); coronary vascular disease variables (bifurcation site, chronic total occlusion (CTO), angulation of diseased vessels, calcification of diseased vessels, type of diseased vessels, and location of diseased vessels).

Among them, e’ wave velocity, history of renal insufficiency, left ventricular mass index, and apolipoprotein A are variables that we did not find in the data sets of other literature [13, 14], but actually have some influence on ACS. Renal insufficiency will activate the renin-angiotensin system (RAAS) and sympathetic nervous system, aggravate cardiac insufficiency, and affect long-term prognosis and survival. The left ventricular mass index is used as a diagnostic index related to left ventricular diastolic dysfunction, and thus is related to long-term prognosis and survival. Low apolipoprotein A makes it easy to have high blood lipids, which increases the risk of coronary heart disease and affects survival. The e’ wave velocity is one of the diagnostic indicators of left ventricular diastolic dysfunction.

### 3.4 The overall pipeline procedure

**Fig 1:**
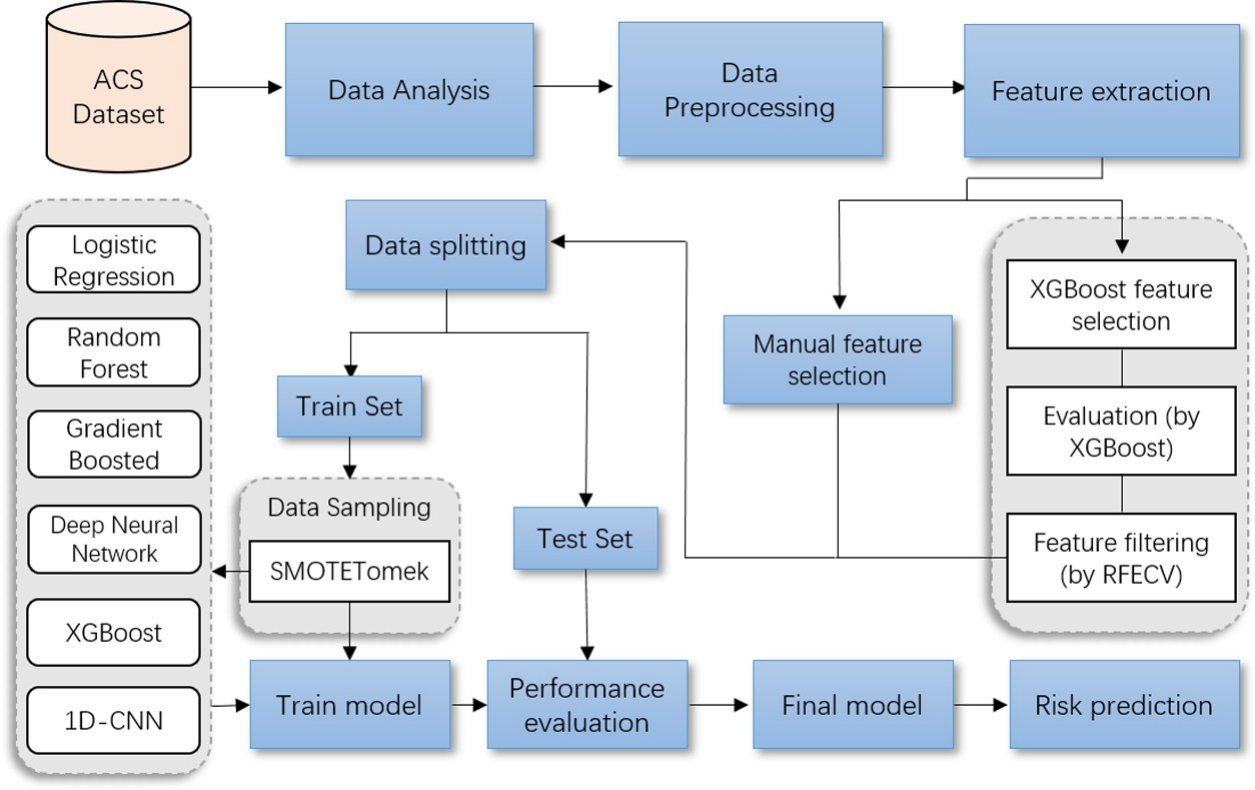
Model development and evaluation pipeline. Process flowchart for visual data processing and model development.

Fig 1 depicts the process from raw data to predictive model development and their evaluation process to determine a subject’s risk probability of developing ACS. The pipeline consists of three distinct operational stages: 1) data mining and modeling; 2) model development; and 3) model evaluation. Our ACS model development and validation strictly follows the process of this pipeline. First, we extracted experimental data from ACS patients discharged after PCI, statistically analyzed the association of each variable, and then preprocessed the dataset and coded categorical variables. Second, we screened ACS key features using a hybrid feature selection method and divided the training cohort (60%) and test cohort (40%) into different proportions by experimental data. Third, we use the SMOTETomek hybrid sampling method to deal with class imbalance in the experimental dataset. We then applied five widely used machine learning algorithms and convolutional neural network model for predicting the postoperative risk of ACS. Finally, we evaluate the performance and comparison results of all models.

### 3.5 ACS Hybrid Feature Selection (XGBoost + RFECV)

XGBoost [30] is an optimized and improved gradient boosted tree system, and this paper adopts XGBoost for the implementation of the gradient boosted tree model. Due to the high dimensionality and sparsity of ACS features, we consider tree models to build interpretable feature branches. To maximize the gain value of the entire tree after splitting, each layer chooses a feature split point as a leaf node while developing the tree model. Accordingly, the more times a feature is divided, the more value it adds to the overall tree and the more significant it becomes. The tree growth process is a process of heuristically searching for feature subsets, so tree models are often combined with feature importance ranking to achieve interpretable feature learning.

After the gradient boosted tree (GB) is created, it is relatively straightforward to get the importance score of each attribute. Similar to GB, XGBoost can also quickly and efficiently obtain the importance of each feature during the node splitting process of all established trees. where *L* represents the loss function, *f*_*t*_ represents the *t*-layer tree, and *Ω*(*f*_*t*_) is the regularization term. The second-order Taylor series of *L* at the *t*-th iteration is shown in Eq 1.

$$L^{\left(t\right)}=\sum_{i=1}^{k}\left[l\left(y_{i},y_{i}^{\left(t-1\right)}\right)+g_{i}f_{t}(x_{i})+\frac{1}{2}h_{i}f_{t}^{2}(x)\right]+\Omega (ft)$$ (1)

Here, *g*_*i*_ and *h*_*i*_ represent the first and second order gradient statistics. When we train XGBoost, we usually use *Gain* to determine the best split node.

$$Gain=\frac{1}{2}\left[\frac{{\left(\sum_{i\in I_{L}}g_{i}\right)}^{2}}{\sum_{i\in I_{L}}h_{i}+\lambda }+\frac{{\left(\sum_{i\in I_{R}}g_{i}\right)}^{2}}{\sum_{i\in I_{R}}h_{i}+\lambda }+\frac{{\left(\sum_{i\in I}g_{i}\right)}^{2}}{\sum_{i\in I}h_{i}+\lambda }\right]-\gamma$$ (2)

where *I*_*L*_ and *I*_*R*_ represent the sample nodes on the left and right after segmentation, respectively (see Eq 2). *I* denotes the intersection of *I*_*L*_ and *I*_*R*_; *λ* and *γ* are penalty parameters. *Gain* represents the gain score after each split of the tree. The gain is not a calculated split, but a measure of the reduction in impurities in the actual node, which is the average gain of all splits using that feature. The average gain is calculated by dividing the overall gain for all trees by the total number of feature splits. The average gain is used to determine the final feature significance score. After the XGBoost model is constructed, the feature ranking based on the importance of gain can be obtained, including all feature importance scores that may affect the results [31]. This score indicates the usefulness or value of each feature in building the model’s boosted decision tree. For recursive feature elimination (REF), the large drop in accuracy indicates that this feature is highly relevant and useful.

#### 3.5.1 Feature selection process

Our proposed hybrid feature selection method is shown in Algorithm 1. The reason why this paper does not directly use the above feature selection method is as follows: Among the existing feature selection methods, tree-based ensemble learning algorithms (such as random forests, gradient boosting trees, etc.) can automatically generate feature importance rankings according to Gini importance (mean reduction in impurities, MDI) after model estimation [24]. This feature selection strategy is characterized by fast execution but randomness, which is easy to cause the existence of a small number of redundant features. Whereas for recursive feature elimination (RFE) where permutation importance (Mean Precision Degradation, MDA) is used, the best feature combination can be found by recursively removing the least important features to improve generalization performance [32]. It is more accurate to iteratively select features using RFE with cross validation (RFECV). But the defect of RFECV feature selection is that it takes a lot of time in the search process. And we hope to make up for the weaknesses of each algorithm by combining these two FS methods.

**Fig 2:**
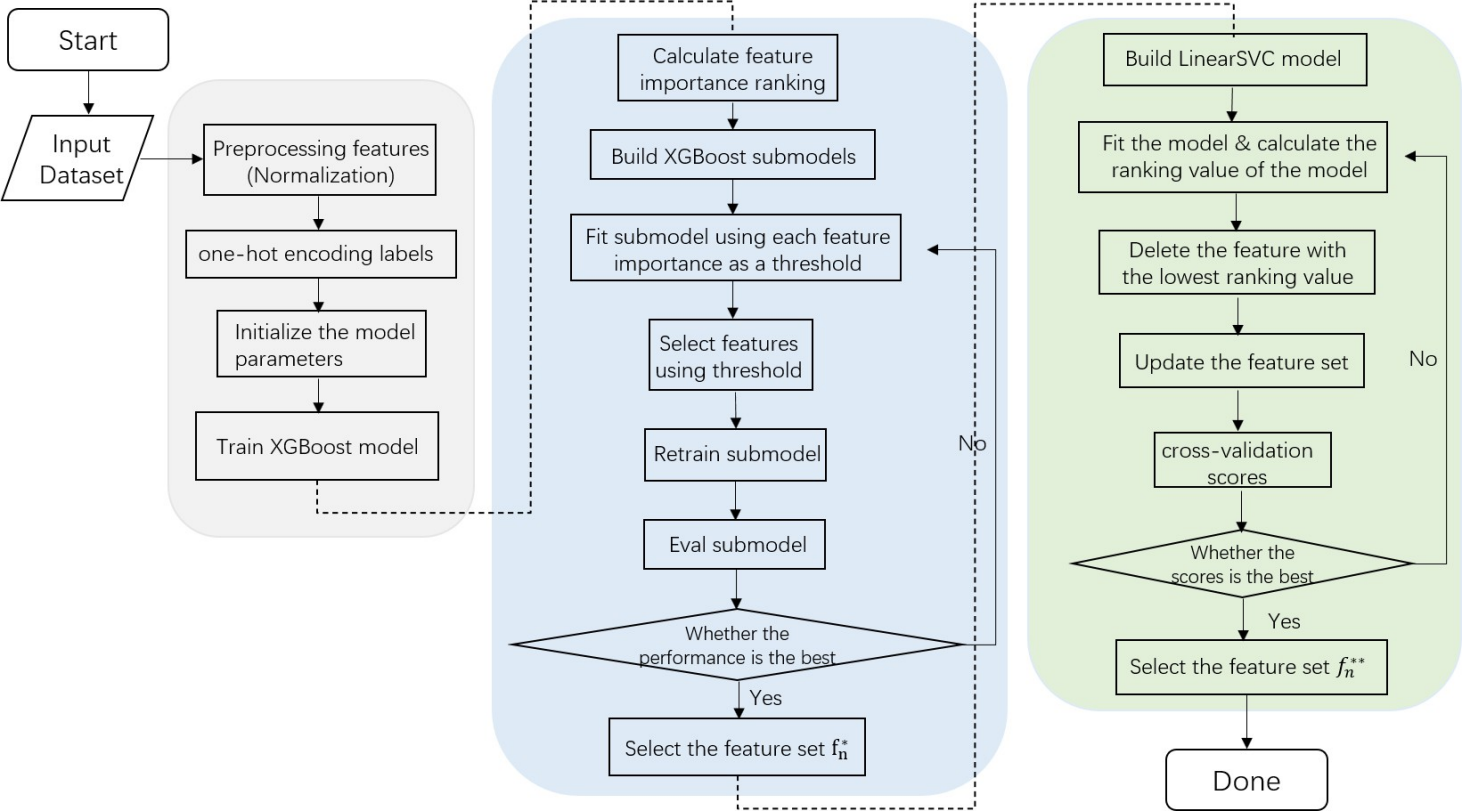
Flowchart of the hybrid feature selection method.

Fig 2 shows the process of hybrid feature selection proposed in this paper. The process first filters a set of relatively important features based on XGBoost. Where the threshold {*t*_*n*_} represents the possible value of the importance score (*r*_*n*_) of the calculated feature, and the range is [0, *r*_*n*_]. For each threshold *t*_*i*_, there is a set of feature subsets {*f*_*i*_} corresponding to it. By fitting a sub-model at each threshold *t*_*i*_, the performance of the model at that threshold is evaluated. Then compare all the evaluation results and select the *t*_*i*_ with the best performance, and then the feature set filtered based on the gradient tree FS can be initially obtained. The next step is to find the optimal subset from the filtered features using recursive feature elimination with cross validation (RFECV). This reduces a lot of unnecessary time compared to using RFECV directly while also improving the quality of the features being screened. Therefore, this hybrid feature selection method can remove redundant and irrelevant features at the expense of a small amount of accuracy, so as to obtain efficient and streamlined optimal features as much as possible.

#### 3.5.2 Algorithm

**Algorithm 1:** Proposed Hybrid Feature Selection

1. **Input:** training set $S={\left\{(f_{n},y_{n})\right\}}_{n=1}^{N}$, *N* is the amount of data, *Ω* is the entire feature set;
2.
**Feature selection using XGBoost:**

3. Fit the XGBoost model with all features {*f*_*n*_};
4. Feature importance ranking and partition threshold to select features:
  - Calculate the relative importance score {*r*_*n*_} of *Ω* and sort in descending order;
  - Filter features by thresholds;
    1. Define threshold *t*_*n*_ = {0,0.001,…,*r*_*n*_};
    2. **for** every {*t*_*i*_}, **do:**
      - Remove all features *f*_*i*_ with *r*_*i*_ < *t*_*i*_;
      - Get the subset with remaining features *S*_*i*_ = *Ω* -*f*_*i*_;
      - Retrain the subset *S*_*i*_ using the XGBoost model;
      - *A*_*i*_ Obtain the current validation model AUC evaluation performance *A*_*i*_;
    3. **end for**
    4. Select the threshold τ_i_ and the number of features n under the best evaluation performance A_i_;
    5. Select the final feature set $\left\{{\text{f}}_{n}^{*}\right\}$ according to the threshold *τ*_*i*_ and the number of features n;
5.
**Feature selection using RFE-CV:**

6. Recursive feature elimination selects the optimal set:
  - N is the number of feature sets $\left\{{\text{f}}_{n}^{*}\right\}$, C is the number of k-fold cross-validation;
  - **for** every i = N, …, 2, **do:**
    1. **for** every j = 1, …, i, **do:**
      - Remove the feature ${f_{j}}^{\left(i\right)}$ to obtain a subset with remaining features $S_{j}\text{'}=\{{\text{f}}_{n}^{*}\}-f_{j}$;
      - Retrain the subset $S_{j}\text{'}$;
      - Cross-validation to obtain the current Accuracy assessment performance *Acc*_*j*_;
    2. **end for**
    3. Select the best evaluation performance {*Acc*_*j*_}, and eliminate the feature $f_{{}_{j}}^{\left(i\right)}$;
    4. Retain i-1th important features, obtain subset $S_{i}\text{'}$ and evaluate performance *Acc*_*i*_;
  - **end for**
  - Compare the best evaluation performance {*Acc*_*i*_}, and select the final feature set $\left\{{\text{f}}_{n}^{**}\right\}$ with n features;
7. **Output:** Important features after feature selection $\left\{f_{n}^{**}\right\}$.

*3*.*5*.*2*.*1*. *Time complexity*. The number of known data is *N*, the number of features is *D*, the number of spanning trees is *K*, the depth of the tree is *L*, and the number of threshold iterations is *T*. The complexity of pre-sorting for global features is *O*(*DN* log *N*); the complexity of building *K* trees is *O*(*KLND*); the complexity of sorting importance scores is *O*(*D* log *D*); selecting features after iterating over all thresholds The complexity is *O*(*T*(*D* + *DN* log *N* + *KLND*)). The time complexity of RFE-CV feature selection is *O*(*D*^3^): Finally, the total time complexity of XGBoost and RFE-CV feature selection is: *O* (*DT* + (1 + *T*)*DN* log *N* + (1 + *T*)*KLND* + *D* log *D* + *D*^3^)).

### 3.6 Medical data imbalance

Our final ACS dataset showed a data class imbalance between the patients’ survival and death groups. In practical medical problems, especially data samples for binary classification problems, real data often produces missing values, including values related to privacy issues, incomplete data extraction, etc. This will result in an uneven ratio between normal and abnormal samples. This imbalance can lead to biased model learning performance, allowing the model to learn only a small amount of information from data with low proportions. Generally, data sampling techniques to deal with data imbalance can be divided into three categories: oversampling, undersampling, and hybrid methods [33, 34]. BorderlineSMOTE [33] is a typical oversampling technique. Since the survival group to death group ratio in this study is around 23:1, we use the SMOTETomek [35] hybrid strategy to address the issue of class imbalance when training the prediction model. SMOTETomek is a common hybrid sampling strategy that is used to balance the class size impact by increasing the number of minority classes while lowering the number of majority classes. After adding new artificially synthesized minority class samples using the SMOTE technique, the majority class data is reduced using the Tomek Links [36] technique. Better categorization is achieved by eliminating class overlap via Tomek Link, which enables all nearest neighbor samples to belong to the same class.

### 3.7 ACS interpretable machine learning model

In our study, we utilized five supervised learning models to classify high-risk patients—logistic regression (LR) [37], random forest (RF) [38], gradient boosted tree (GBDT) [39], deep neural network (DNN), and Extreme Gradient Boosting (XGBoost) [30]—were used to predict research results. The implementation of the DNN model is a multilayer perceptron (MLP), which consists of multiple fully connected network layers. In addition, we also use a one-dimensional convolutional neural network (1D-CNN) model [40], which works well in sequence models, natural language processing (NLP), etc. Among them, logistic regression and tree models are commonly used interpretable models. Linking the feature representation learned by the model with specific medical prior knowledge is beneficial to help domain experts understand the model’s decision-making process. This interpretable knowledge is introduced into the process of model design and modification through feedback to improve the performance of the model in medical scenarios.

### 3.8. Ethics approval

This research was approved by the Institutional Review Board (IRB) of Fujian Medical University Union Hospital (Approval number: 2021KJCH082). Interviews were conducted following confirmation of informed consent, which was recorded verbally prior to the interview questions. This consent process was approved by the ethics committee. H.Z.L. had access to information that could identify individual participants during or after data collection.

## 4. Experimental results and analysis

### 4.1 Data processing

The data processing of ACS aims to perform operations such as data cleaning, data transformation, missing value filling, and redundant data deletion on the original data. This ensures the quality of the data, so that the accuracy of the results can be obtained during data analysis and large deviations in predictions can be avoided.

1. Data cleaning. There is a lot of redundant and confusing data in the original phenotype data. We manually screened important factors with the advice of doctors, and eliminated characteristic factors that had little impact on the classification results. This includes: name, hospital number, date of surgery, and data records of some surgical operations. We initially selected 87 relatively important features. According to the related research on high risk factors for ACS [5], we also set basic characteristics in this dataset, including age, gender, BMI, smoking status, history of diabetes, and history of hypertension.
2. Data deduplication. We select the patient ID number as the unique attribute, delete the data whose ID number does not exist, and retain 5764 valid records. Then, the ID number attribute is deduplicated, and the patient data record at the latest time point (take the last record as an example) is retained. There are 4562 pieces of data remaining.
3. Handling of missing values and outliers. First, all patients whose information loss rate exceeds 80% are filtered, and the patient data with relatively complete information is retained, with a total of 2702 pieces of data. Then we sequentially process these 87 columns of data features, using the interquartile range to detect outliers and setting the default value to be the outlier. Then, the upper and lower limits of the standard are set for the indicators of each feature to constrain outliers. For outliers out of bounds, the upper and lower bounds under the current column properties will be used instead.
4. Data conversion. The multi-dimensional features of the dataset are discretely distributed, so it is necessary to uniformly standardize the data. To keep each feature in the range [0,1] with a mean of 0 and a variance of 1, we use the Z-score normalization method ($Z=\frac{X-\bar{X}}{\sigma }$).

### 4.2 Statistical analysis

From the information collected in Table 1, we observed the statistical distribution of data between the survival and death groups from different perspectives. Among the clinical features with a p value of less than 0.001 were age, gender, history of renal insufficiency, left ventricular ejection fraction, statin, spironolactone, aspirin use, and angulation of diseased vessels. The differences in these variables were statistically significant.

In the gender distribution of the ACS patient population, the number of male patients was 2207 (81.7%), 2127 were at low risk, and 80 died (high risk); the number of female patients was 495 (18.3%), 455 were at low risk, and 40 died. In the BMI distribution of the ACS patient population, the median BMI in the low-risk group was 24.0, the upper quartile (Q3) was 31.3, the lower quartile (Q1) was 17.5, and the number of outliers was 48. In the high-risk group, the median BMI was 24.0, Q3 was 27.3, Q1 was 20.1, and the number of outliers was 10. In the age distribution of ACS patients, the median age of the low-risk group was 65, with the largest number of patients between the ages of 57 and 71, showing a dense distribution; The median age of the high-risk group is 72, and 64–78 years is the high-frequency period of all-cause mortality, and the number of deaths in this range is higher than that in other ranges; In the distribution of lifestyle habits (smoking, history of diabetes, and history of hypertension) in the ACS patient population, the number of smokers in the low-risk group was 1129 (43.9%), and the number of smokers in the high-risk group was 42 (38.5%); the low-risk group had diabetes, 748 people (29.4%), and 46 people (42.6%) in the high-risk group; 1473 (57.2%) people in the low-risk group had hypertension, and 69 (65.1%) in the high-risk group.

**Fig 3:**
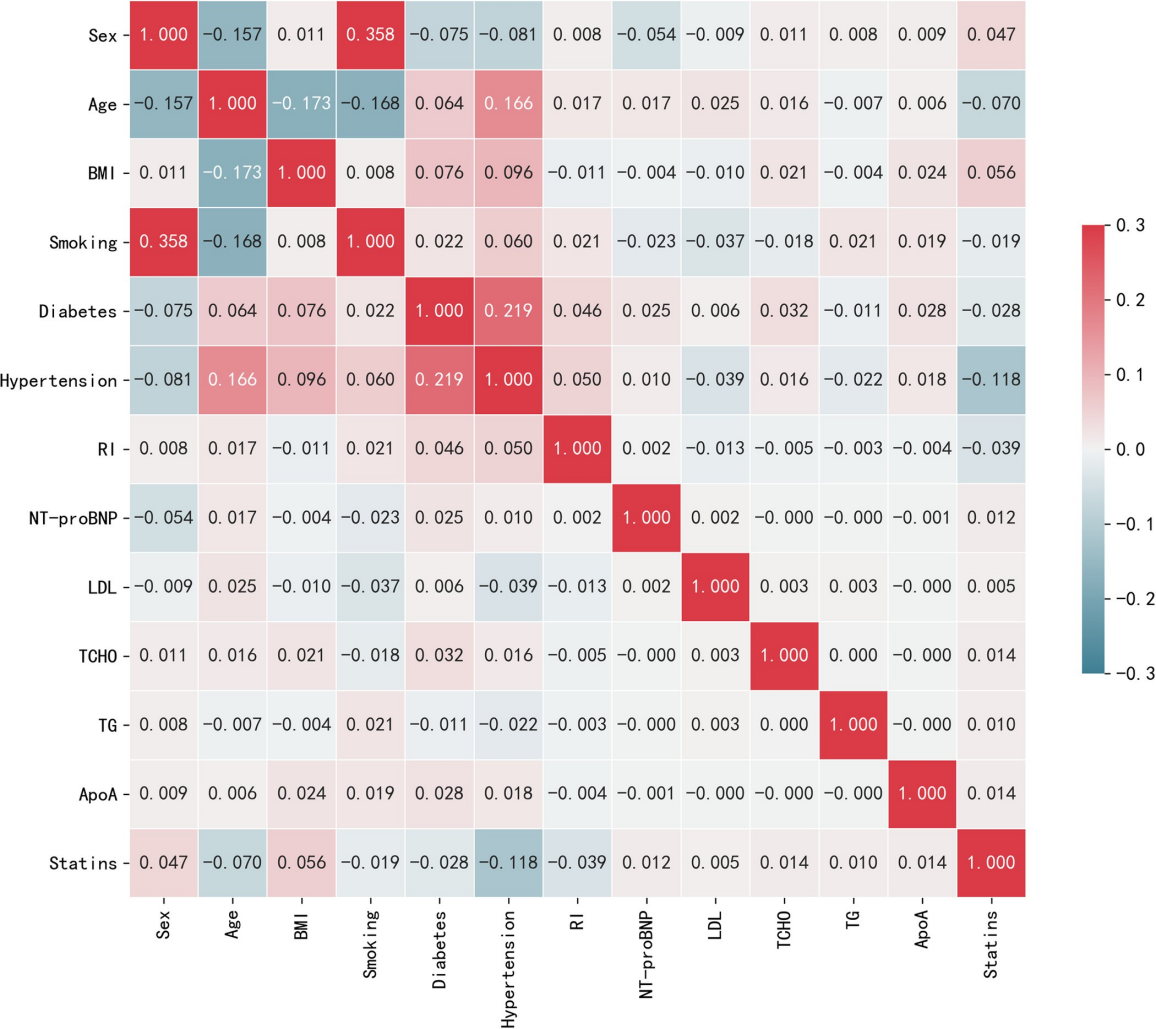
Interpretability of the correlation heatmap.

The main predictors varied by study results. Fig 3 interpretably illustrates the correlation heatmap of selected features (top 15). The correlation heatmap drawn observes the correlation of multiple features with each other from the data table that is effective for prediction. The darker the color, the higher the correlation coefficient, that is, the larger the value in the graph.

### 4.3 Performance evaluation

In the final stage of the pipeline shown in Fig 1, the scores of the models are compared to evaluate their performance in risk prediction. Binary model evaluations (case and non-case) are based on sensitivity ($\frac{TP}{TP+FN}$) and specificity ($\frac{TN}{TN+FP}$), where TP, FP, TN, and FN represent true positives, false positives, true negatives, and false negatives, respectively. False positives are observations that are predicted to be positive but are not actually positive. A false negative is a result that is predicted to be a counterexample but is not actually a counterexample. Area under the curve (AUC) and receiver operating characteristic (ROC) were used to understand the relationship between the two performance variables. The F1-score [41] is the harmonic mean of precision and recall, which allows comparing the performance of different models in identifying true disease predictions when compared to false positives. F1 = $\left(\frac{2*precision*recall}{precision+recall}\right)$, where precision = $\left(\frac{TP}{TP+FP}\right)$ and recall = $\left(\frac{TP}{TP+FN}\right)$. In our experiments, we use macro precision and macro recall as evaluation criteria.

### 4.4. Hyperparameter tuning

In order to develop accurate predictive models and minimize classification errors for all-cause death in ACS patients, we optimized several important parameters of the applied ML algorithm using hyperparameter tuning. Table 4 shows the hyperparameter tuning of the applied ML algorithm. Moreover, in order to obtain the optimal hyperparameter value, we select the final value by fine-tuning. To ensure fair results for the original dataset ML experiments, we all use the same parameters as the FS and mixed sampling experiments. All experiments were performed on a fixed random number seed of 21. For the deep model, the training batches are all 300 epochs and the learning rate is 0.01. The model’s optimizer uses adam.

**Table 4:** Hyperparameter tuning of machine learning algorithms.

Algorithm	Parameter Configuration	Value
Logistic Regression	{penalty, solver, C, max_iter}	{‘l2’, ‘liblinear’, ‘1.0’, ‘100’}
Random Forest	{criterion, n_estimators}	{‘gini’, ‘100’}
Gradient Boosting	{criterion, n_estimators, learning_rate}	{‘friedman_mse’, ‘100’,’0.1’}
XGBoost	{booster, gamma, n_estimators, learning_rate}	{‘gbtree’, ‘1’, ‘100’,’0.1’}
Deep Neural Network	{epoch, batch_size, activation, loss, network layer}	{‘300’, ‘100’, ‘relu’, ‘binary_crossentropy’, [12-50-50-50-1]}
1D-CNN	{epoch, batch_size, activation, loss, network layer}	{‘300’, ‘100’, ‘relu’, ‘categorical_crossentropy, [25–32–32–64–64–64–64–2]}

### 4.5 Feature selection

#### 4.5.1 Feature importance ranking and recursive elimination

The data set obtained after preliminary data processing needs further feature selection. We evaluated the significance of each XGBoost model feature and ranked them in descending order of significance to identify the key predictors of each research outcome in the patient group. The importance score is calculated by node splitting, that is, the influence of the corresponding feature on the result. Then, we pick a fixed number of features based on a threshold (t). After each iteration, the top-ranked features with scores higher than t are selected to be added to the feature set.

**Fig 4:**
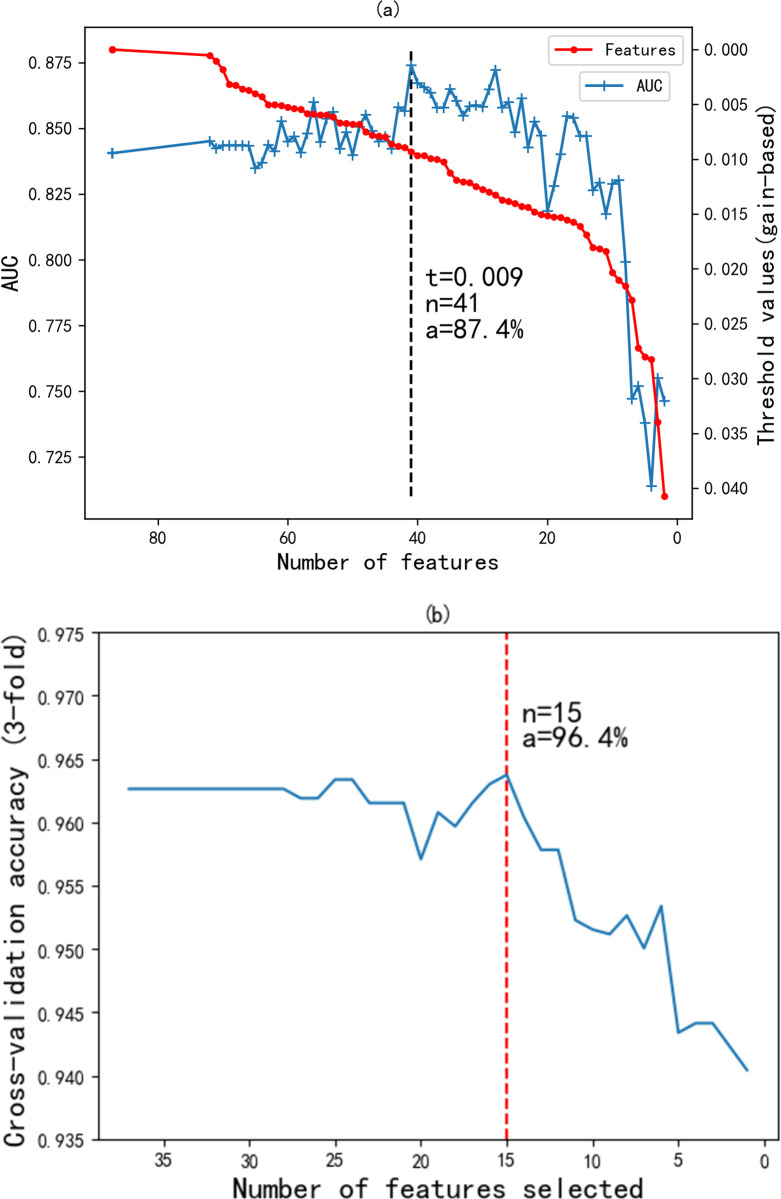
Hybrid feature selection. (a) feature filtering by relative importance ranking; (b) feature combination selection by RFECV.

Fig 4A depicts the feature filtering of the relative importance ranking. As the threshold t increases, the number of selected features n is decreasing. By observing the AUC performance under different feature-thresholds, we found that the AUC of the XGboost evaluation model before feature selection (n = 87) was 84.1%. The AUC performance did not change as the threshold was increased to 0.001 (n = 72), indicating that the 15 features in between had no effect on the results. When the threshold is 0.009 and the number of features is 41, the AUC achieves the best effect of 87.4%; when the threshold is 0.009 and the number of features is 15, the AUC is 84.7%.

For the better feature subset obtained in XGBoost, we filtered a total of 31 impurity features. In order to further obtain fewer features and better performance, we also need to eliminate features according to the 3-fold cross-validation RFE method (see Fig 4B). By model retraining and iteratively pruning the features in the current set with the least importance, the optimal feature combination is chosen from the filtered features in the RFE process. Recursively eliminating the less correlated features from the 41 features yielded the best-performing combination with 15 features, achieving 96.4% accuracy. Compared with the original features (n = 87), the number of features is reduced by 72, and the AUC is increased by 0.6%, while the AUC is only decreased by 2.7% compared to the best effect (n = 41). Considering the trade-off between complexity and performance, we therefore recommend choosing the 15 factors with the optimal number of features.

#### 4.5.2 Interpretability feature contributions

In addition to the important factors calculated by feature selection, we also manually added surgery-related factors for ACS, which were combined into the total feature set. The final result is 25 variables with important weights to the classification results (see Section 4.1.3), which provide most of the overall importance weights.

**Fig 5:**
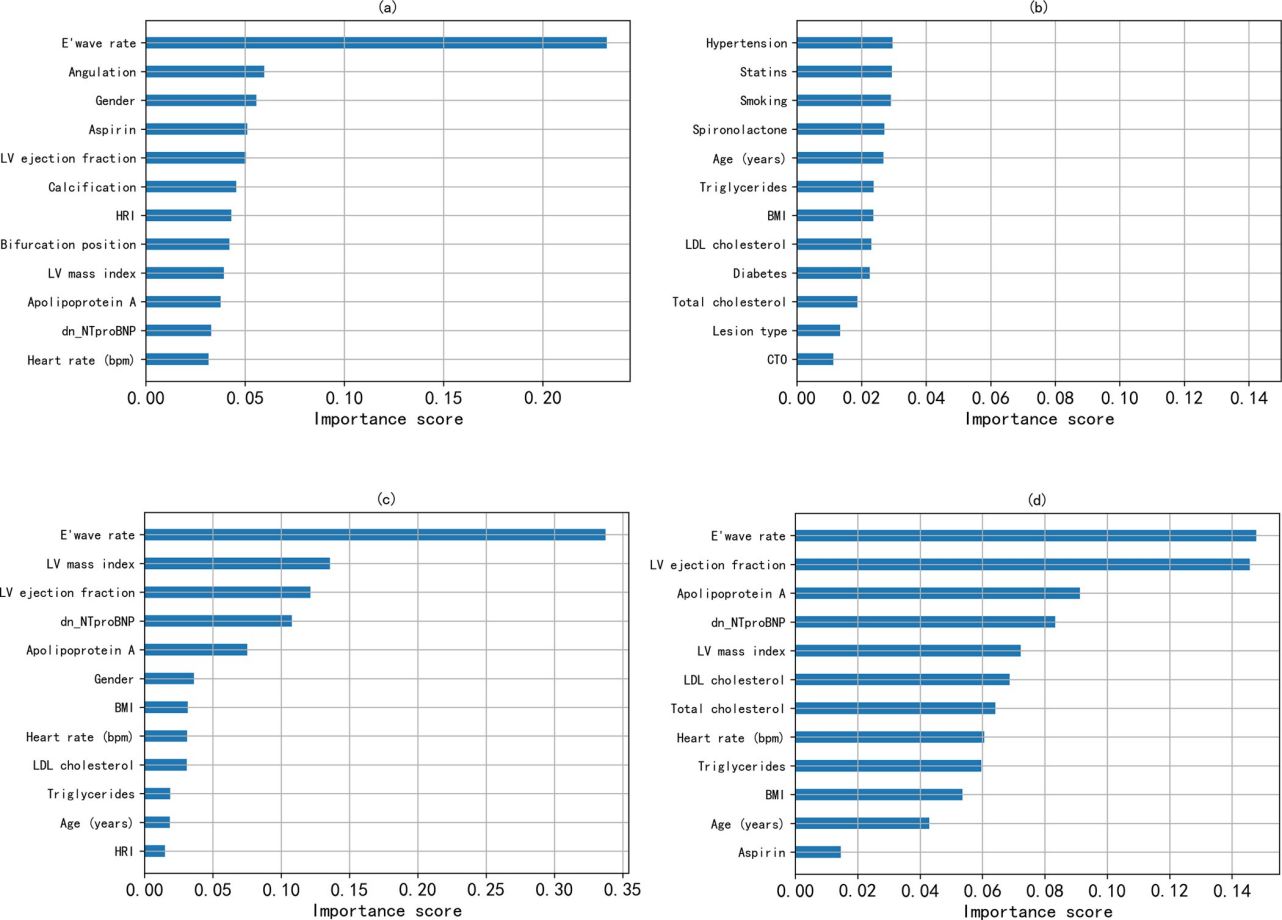
Feature importance scores calculated by information gain. (a) ranking of the XGBoost top 12 ACS feature contributions; (b) ranking of the XGBoost bottom 12 ACS feature contributions; (c) ranking of the GB model’s feature contributions; and (d) ranking of the RF model’s feature contributions.

Importance scores interpretably demonstrate the degree to which features are associated with ACS survival-death, with each feature contributing differently (see Fig 5). And the e’ wave velocity explained the largest contribution to the prediction, exceeding 0.2. This suggests that this feature makes a diagnosis of ACS a 2-fold higher chance of death than survival when other features are held constant. Finally, it can be found that e’ wave velocity, target vessel angulation, gender, left ventricular ejection fraction, etc. play an important role in all-cause mortality.

### 4.6 Model comparison results

Tables 5 and 6 show the prediction results of different machine learning models on the ACS dataset. The results in Table 5 are the performance only before or after data preprocessing. The results in Table 6 are the performance after XGBoost feature selection and SMOTETomek hybrid sampling based on Table 5. The time performance comparison of the model in Table 10 is the mean result calculated by 5 experiments, and the value in (*) is the variance result of the experimental data.

**Table 5:** Evaluation performance of the original ACS prediction model before/after preprocessing.

Model	Accuracy	AUC	Precision	Recall	F1 score
Logistic Regression	96.9	79.8	66.3	54.3	56.4
Random Forest	97.2	78.1	86.1	52.3	56.3
Gradient Boosting	96.5	78.9	59.8	54.1	55.6
XGBoost	**97.2**	80.3	79.9	53.7	56.1
Deep Neural Network	96.1	68.5	57.0	53.9	54.9
1D-CNN	97.1	58.3	70.9	52.9	54.6
Logistic Regression	96.1	87.1	79.5	72.8	75.7
Random Forest	97.1	**90.7**	**94.5**	71.5	78.6
Gradient Boosting	97.1	89.3	89.4	**75.2**	**80.6**
XGBoost	96.9	89.8	90.6	70.4	76.8
Deep Neural Network	95.1	80.3	72.4	69.5	70.8
1D-CNN	96.5	76.8	82.5	73.9	77.5

**Table 6:** Evaluation performance of the ACS prediction model after feature selection and hybrid sampling.

Model	Accuracy	AUC	Precision	Recall	F1 score
Logistic Regression	83.0	89.9	58.8	80.8	60.3
Random Forest	98.6	**99.9**	89.0	98.3	93.1
Gradient Boosting	96.6	97.3	79.7	88.9	83.5
XGBoost	98.8	99.8	90.7	97.5	93.8
Deep Neural Network	98.1	99.6	85.4	**99.0**	90.9
1D-CNN	96.8	99.8	79.7	98.3	86.4

## 5. Discussion

In this study, we developed and tested a machine learning-based risk model to predict all-cause mortality (cardiac and non-cardiac) 1 year after discharge using data from 2702 ACS patients discharged after PCI. Thrombolysis in Myocardial Infarction (TIMI score) and Global Registry of Acute Coronary Events (GRACE score) scores are currently the most widely used predictive scoring models in clinical practice, but none of the study populations at that time received the current standard treatment. Our included study population received standard care and revascularization surgery, and surgery-related indicators were added to the predictors, thus providing high accuracy in detecting the risk of all-cause mortality after acute coronary syndrome (ACS).

For logistic regression (LR), the Accuracy, AUC, and F1 scores before feature selection are 96.1%, 87.1%, and 75.7%. After feature selection (FS) and hybrid sampling (HS), AUC performance improved by 2.8%, and the rest of the performance decreased by 13.1% and 26.8%, respectively; For random forest (RF), the Accuracy, AUC, and F1 scores before feature selection are 97.1%, 90.7%, and 78.6%. After FS and HS, the Accuracy performance improved by 1.5%, the AUC performance improved by 9.2%, and the F1 performance improved by 14.5%; For gradient boosting (GB), the Accuracy, AUC, and F1 scores before FS are 97.1%, 89.3%, and 80.6%. After FS and HS, the AUC performance is improved by 8.0%, the F1 performance is improved by 2.9%, and the Accuracy performance is decreased by 0.5%; For deep neural networks (DNN), after FS and HS, the Accuracy performance is improved by 3.0%, the AUC performance is improved by 19.3%, and the F1 performance is improved by 20.1%; for extreme gradient boosting (XGBoost), the Accuracy performance is improved after FS and HS by 1.9%, the AUC performance increased by 10.0%, and the F1 performance increased by 17.0%. For one-Dimensional Convolutional Neural Network (1D-CNN), after FS and HS, the Accuracy performance is improved by 0.3%, the AUC performance is improved by 23.0%, and the F1 performance is improved by 8.9%.

Through comparison, it is found that, except for the obvious decline in the performance of LR, the performance of other models after XGBoost feature selection and SMOTETomek hybrid sampling has been improved to a certain extent. This is the shortcoming of LR in dealing with nonlinear problems. Table 7 shows the AUC comparison results of different feature selection methods. We chose the chi-square test, gradient boosting, and recursive elimination feature selection method and this paper for comparison experiments. In order to ensure fair results, we performed four feature selection experiments on the 87-dimensional ACS dataset, respectively, and finally retained the same number of features (25) across both datasets. We can observe that the performance of the method proposed in this paper is generally better than the other FS methods mentioned above, except that the performance of the LR and GB models is slightly worse. Therefore, the ACS features screened by the FS method in this paper are effective.

**Table 7:** AUC comparison results for different feature selection methods.

Model	LR	RF	GB	XGBoost	DNN	1D-CNN
Chi-square test	79.9	79.5	82.5	77.9	74.4	75.3
GBDT-FS	78.5	83.8	83.3	83.1	66.5	70.0
RFE-FS	82.2	84.5	85.0	84.1	67.7	74.5
RFECV-FS	**87.2**	84.7	**88.0**	84.8	73.4	74.9
Proposed	87.1	**88.4**	86.2	**85.0**	**78.7**	**77.2**

We found that the model performance degrades after adopting FS under imbalanced data, so we choose the sampling technique. Table 8 shows the AUC comparison results of different sampling methods. We chose undersampling, oversampling, and BorderlineSMOTE for comparative experiments. From this, we can observe that SMOTETomek has the best AUC performance in terms of improving the prediction performance of the model after FS. Therefore, we chose SMOTETomek as the sampling method to be effective in this paper.

**Table 8:** AUC comparison results for different sampling techniques.

Model	LR	RF	GB	XGBoost	DNN	1DCNN
UnderSampler	88.0	94.7	82.5	92.5	88.1	87.3
OverSampler	84.1	87.6	82.0	80.9	78.9	77.2
BorderlineSMOTE	87.1	99.1	95.7	98.6	98.9	98.8
SMOTETomek	**89.9**	**99.9**	**97.3**	**99.8**	**99.6**	**99.8**

Table 9 shows the ablation experiments of our method. We investigate the variation in Accuracy of different models after removing (a) FS; (b) hybrid sampling; and (c) FS and hybrid sampling, respectively. It can be observed that after removing FS and SMOTETomek, the Accuracy of most models (RF, XGBoost, DNN, and 1D-CNN) dropped significantly, reflecting the effectiveness of this method; After removing SMOTETomek (only using FS), some models (RF, XGBoost, and DNN) have more Accuracy drops than (c), indicating the importance of FS; After removing FS (using only mixed sampling), the model accuracy works well. However, the problem of too many features has not been solved, and the interpretability is poor, so this article does not adopt it.

**Table 9:** Ablation experiments of the proposed method (accuracy comparison).

Model	LR	RF	GB	XGBoost	DNN	1D-CNN
Proposed	83.0	98.6	96.6	98.8	98.1	96.8
(w/o) FS+SMOTETomek	96.1	97.1	97.1	96.9	95.1	96.5
(w/o) FS	85.6	99.0	98.0	99.3	97.6	98.9
(w/o) SMOTETomek	96.3	97.3	95.7	97.2	95.4	95.7

The results in Table 10 show that the temporal dimension of each model is somewhat reduced after using our hybrid feature selection. Compared with the results of RFE and RFECV in Table 7 and Table 10, it is found that the AUC effect of RFECV is better than that of RFE, but the time performance is far inferior to that of RFE. This means that it requires more time for cross-validation to screen features, thereby improving the predictive performance of the model. Compared with the feature selection method in this paper, both the AUC performance and the time performance are better than the RFECV method. The AUC is close to the latter, and even some models are better than the latter. Therefore, the method in this paper is effective in the process of selecting ACS features. The method in this paper reduces the number of features from 87 to 25, with a reduction rate of 71.3%. And the time performance is between XGboost and RFECV. After removing a large number of irrelevant and unimportant features, we shifted our focus to the main factors of the study. The ROC curve will be plotted below to visualize this change.

**Table 10:** Time performance comparison of ACS prediction models and FS method.

Index	Model	Original time (s)	Time after FS (s)	Improvement (%)
1	Logistic Regression	0.057(±0.0057)	0.030(±0.0022)	47.5%
2	Random Forest	0.770(±0.0343)	0.461(±0.0134)	40.1%
3	Gradient Boosting	1.730(±0.0158)	0.515(±0.0200)	70.2%
4	XGBoost	0.306(±0.0452)	0.144(±0.0101)	53.1%
5	Deep Neural Network	13.855(±0.4814)	13.502(±0.1782)	5.2%
6	1D-CNN	128.373(±3.6955)	70.287(±1.0336)	45.2%
Index	FS Method		Processing time (s)	
7	RFE		7.211(±0.3250)	
8	RFECV		68.607(±0.9883)	
9	XGboost		25.640(±0.2424)	
10	Proposed		38.823(±0.3643)	

**Fig 6:**
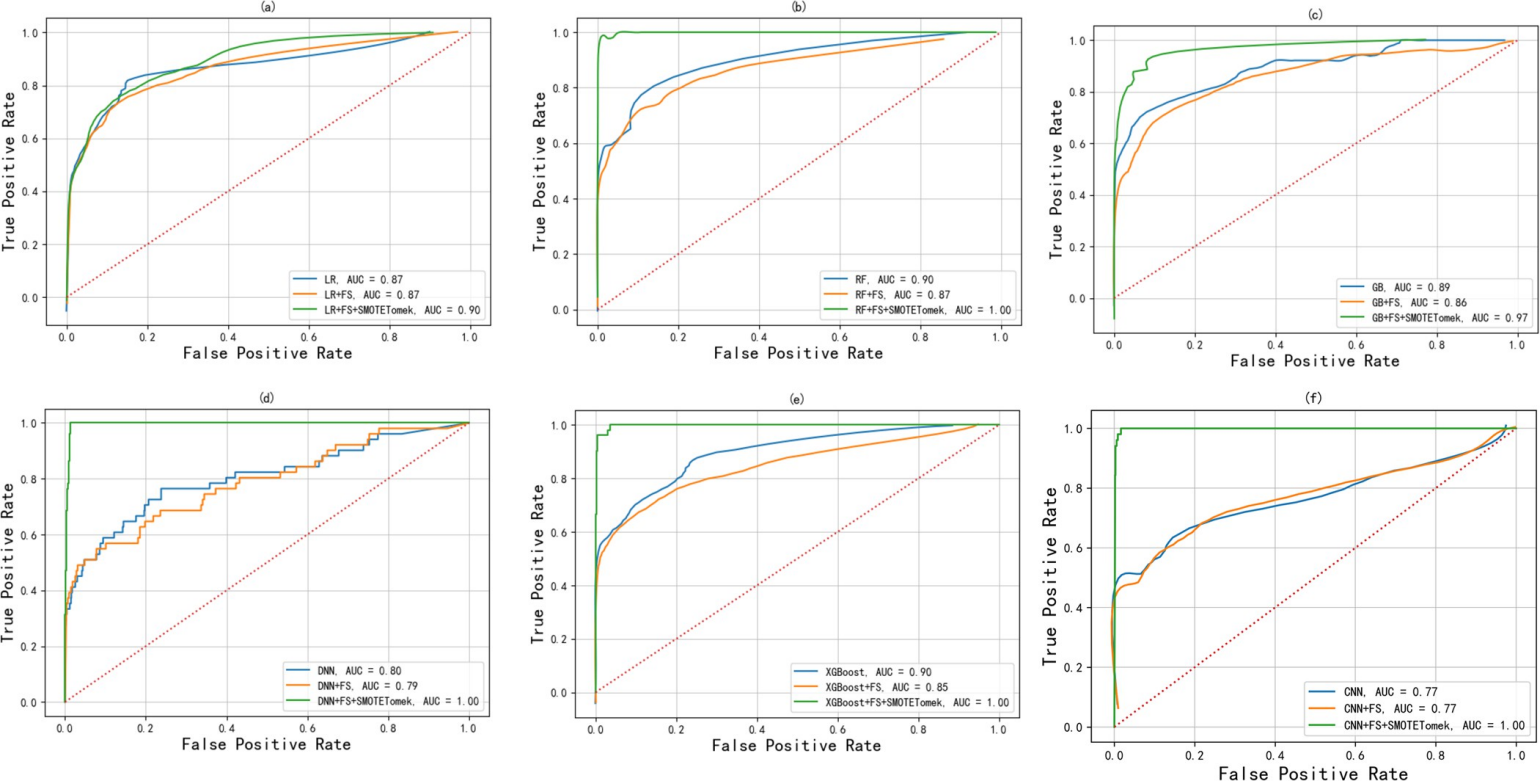
ROC curves of the model under non-feature selection, feature selection, and mixed sampling.

It can be observed from the ROC curves of Fig 6A~6E that, except for the large deviation of the XGBoost (analyzed in the previous section) model (0.85–0.90), the ROC curves of the other models after FS are basically close to the original curve. After SMOTEtomek hybrid sampling, it is found that the area of the ROC curve of most models greatly exceeds the original area. For the ROC curve on LR, the area of the curve after FPR > 0.4 exceeds the original part for the first time; the area of the ROC curve of GB exceeds the original part before FPR 0.7 and fits the original curve at 0.7; for the rest of the models (RF, DNN, and XGBoost), ROC curves reach the optimal level; overall, the method proposed in this paper is remarkably effective.

**Fig 7:**
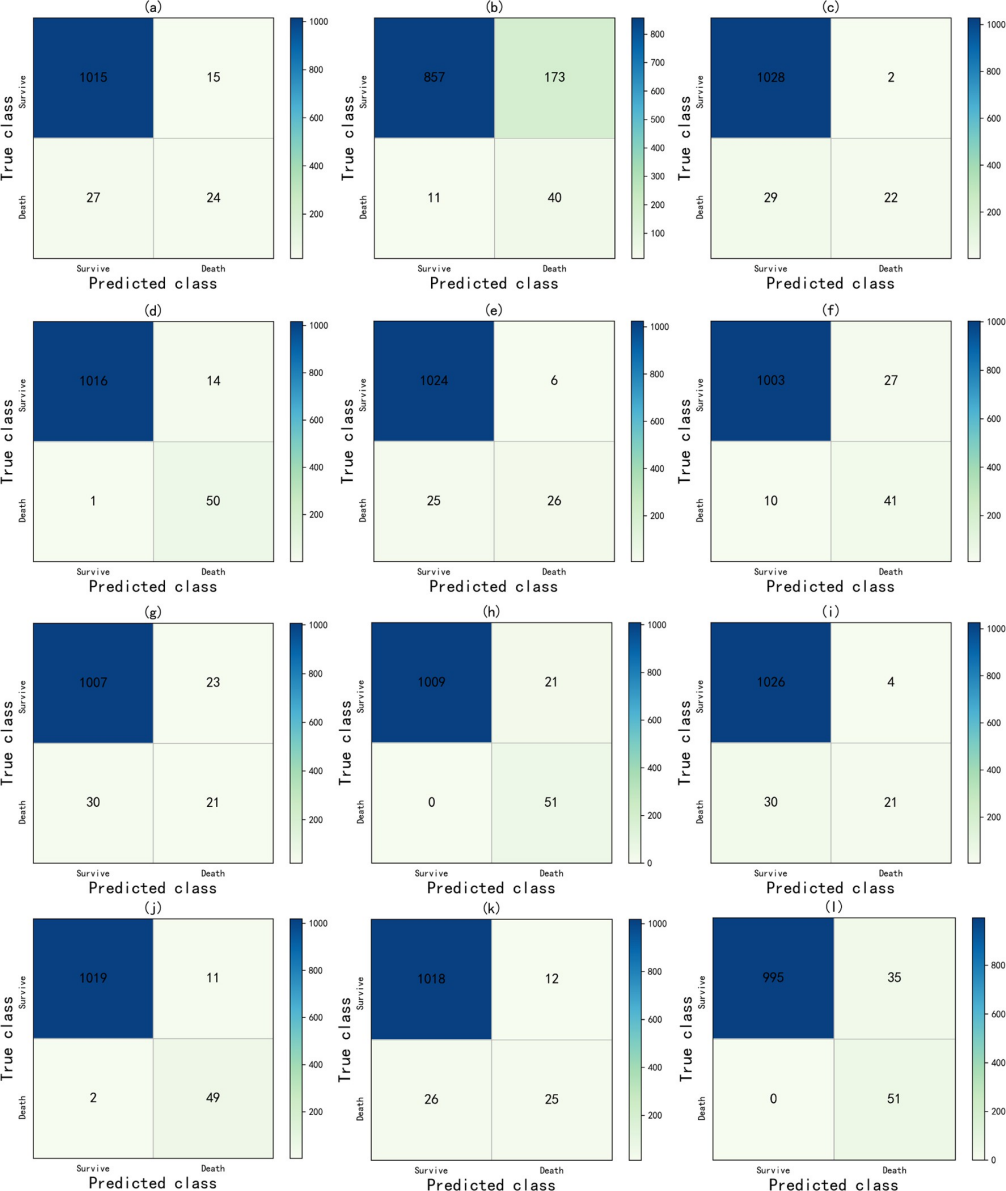
The confusion matrix includes. (1) only each prediction model after data preprocessing, where (a) LR, (c) RF, (e) GB, (g) DNN, (i) XGBoost, (k) CNN; and (2) each prediction model after using feature selection and mixed sampling techniques, where (b) LR, (d) RF, (f) GB, (h) DNN, (j) XGBoost, (l) CNN.

Fig 7 is an explanatory confusion matrix showing the prediction results after only data preprocessing/feature selection and mixed sampling. It assesses the predicted number of survival and death groups in the ACS test cohort. It can be found that after applying the method proposed in this paper, the number of correct predictions of the LR model for the ACS death group in (a)~(b) increased from 24 to 40, but the number of misjudgments in the survival group increased from 15 to 173; In the Fig 7C and 7D, the number of correct predictions of the ACS death group by the RF model has increased from 22 to 50, and the effect is more accurate; In the Fig 7E and 7F, the number of correct predictions by the GB model for the ACS death group increased from 26 to 41, while the number of misjudgments in the survival group increased from 6 to 27; In the Fig 7G and 7H, the number of correct predictions of the DNN model for the ACS death group has increased from 21 to 51, and all the death groups are correctly predicted; In the Fig 7I and 7J, the number of correct predictions of the XGBoost model for the ACS death group increased from 21 to 49, and the number of misjudgments in the survival group increased from 4 to 11; In the Fig 7K and 7L, the number of correct predictions by the one-dimensional convolutional neural network model for the ACS death group increased from 25 to 51, and the number of misjudgments in the survival group increased from 12 to 35.

In conclusion, the combination of the feature selection method and the mixed sampling technique proposed in this paper can predict the death group correctly to the maximum extent under the condition of a low false positive rate, and the performance is better than that of the original group.

## 6. Conclusion

In the actual diagnosis and treatment of acute coronary syndrome, there are a large number of redundant related features, and the research on the features is not yet thorough. So this paper mainly studies a hybrid feature selection (based on gradient boosting trees and RFECV) to assist in screening the ACS for important factors. The method has used XGBoost to filter a set of relatively important features and select the feature set with the best evaluation result at the moment. RFECV is then used again to find the best subset of features from the filtered features. The method of this study successfully extracted 25 factors that play an important role in ACS death from the multi-dimensionally complex medical records (430 factors) in medicine. To better understand the contribution of different features to ACS, this study statistically analyzes the high correlation of features and then uses the interpretability of gradient boosting trees to explain key features.

In the experimental part, this study conducts experiments using six ML models incorporating mixed sampling techniques to predict outcomes for different control groups (survival and death). The experimental results show that we have made breakthroughs in the accuracy and F1 score indicators of each model and have successfully classified the ACS dataset. Therefore, the feature selection method in this paper, combined with the hybrid sampling technique, can provide an automatic identification mechanism for patients at risk of ACS disease. We found that it can improve the predictive performance, and the excellent predictive ability will optimize its application in the diagnosis and treatment of postoperative recurrence while simplifying the diagnosis process. It has significance to a certain extent in the study of ACS.

Our method is very effective for predicting ACS, but there are still some potential limitations in this study, such as the interpretability and performance of this method, which still have a lot of room for improvement. In future work, we will focus on interpretable deep learning models (such as CNN) to improve prediction performance and assist doctors in making timely and correct decisions on ACS diagnosis and treatment. The authors also plan to develop a visual ACS medical decision support system in the future and test the automated system on other ACS datasets.
